# High-Voltage Electrical Burn Requiring Urgent Scalp Reconstruction after Developing a Brain Abscess

**DOI:** 10.3390/ebj5030027

**Published:** 2024-09-03

**Authors:** Elena Blyth, Elizabeth Vujcich, Darryl Dunn

**Affiliations:** 1 The Professor Stuart Pegg Adult Burns Centre, The Royal Brisbane and Women’s Hospital, Brisbane 4029, Australia; elizabeth.vujcich@health.qld.gov.au; 2 Department of Plastic and Reconstructive Surgery, The Royal Brisbane and Women’s Hospital, Brisbane 4029, Australia; darryl.dunn@health.qld.gov.au

**Keywords:** electrical burn, scalp reconstruction, brain abscess, spinal cord injury

## Abstract

Electrical burn injuries to the scalp are at risk of extensive tissue damage and neurological complications. We present the case of a patient who came into contact with a high-voltage power line while cherry picking, resulting in a large full-thickness scalp defect. Early on in his presentation, he developed progressive global weakness which remained relatively static during his admission. An incidental finding of an extradural abscess complicated his management, requiring urgent surgical intervention with definitive tissue coverage. The scalp was reconstructed using a free myocutaneous anterolateral thigh flap. There were no postoperative complications. Following rehabilitation, the patient was discharged home with limited functional recovery. He mobilises independently with a wheelchair and requires full-time carers.

## 1. Introduction

Electrical burns are associated with high morbidity and mortality depending on the organ systems involved [1,2]. They can be classified as low-voltage (<1000 V) or high-voltage (>1000 V), with the latter more likely to produce ischaemic injury and extensive damage to the skin and underlying tissues [1,2].

Electrical energy is converted to heat energy during the course of conduction through the body, the amount of heat dissipated is related to the resistance of different tissues [3]. In high-voltage electrical burns, the resistance at the contact point of the electrical source determines the severity of injury [4]. Blood vessels, nerves, and muscles have the least resistance, in contrast to skin, bone, and fat which are the highest and tend to suffer the most damage [4,5].

The source and ground of the current can predict the pattern and distribution of electrical injury. The scalp is a relatively rare contact point but has the potential to result in large full-thickness defects due to the relative thickness and density of resistance [3,4]. In addition, neurological complications can arise including cerebral, spinal, and peripheral nerve syndromes [4,5]. Direct cerebral injury may result in hemiplegia, aphasia, epilepsy, or cerebellar dysfunction [5]. Spinal cord injury can be classified as immediate or delayed, with symptoms developing days to months following the incident [5]. Motor nerve dysfunction occurs more frequently than sensory loss, likely due to the relative vulnerability of anterior horn cells to ischaemic injury [2,5]. The mechanism for peripheral neuropathy involves axonal damage, producing both motor and sensory deficits [6]

The reconstructive plan for electrical burns of the scalp requires consideration of the size, depth, and location of the defect [2,3]. Management can be challenging with large areas of tissue loss and exposure to the underlying calvarium [3]. The goal is to achieve coverage with well-vascularised tissue while minimising patient morbidity [7]. Considerations also need to be made regarding the calvarium, as necrotic exposed bone requires the removal of the inner and outer table, if they are not viable, to prevent chronic infection. Within the literature, there is only one case report describing an intracranial abscess two months following an electrical burn [8].

Malnutrition is associated with the increased risk and severity of postoperative complications [9]. Optimising the nutritional intake and metabolic reserve is recommended, despite possible delays in surgical intervention [9,10].

We present the case of a high-voltage electrical burn in a 69-year-old male that unexpectedly developed an extradural abscess with an intact calvarium. The surgical implications relating to his immediate management are discussed.

## 2. Case Report

A 69-year-old male presented with electrical burns following contact with a high-voltage power line while picking cherries in a fruit picker. He lost consciousness for an unknown period of time before waking up and driving himself home.

The patient sought medical attention two days later as he could not see the extent of injury due to facial and orbital oedema.

He was taken to a regional Hospital to facilitate transfer to a tertiary centre with a specialised burns service.

Significant background medical history is as follows:

- Low BMI—exclusive fruitarian diet.
- Laparoscopic partial gastrectomy (7 September 2022)—T4N0R0 GIST—Adjuvant imatinib from October to December 2022 (patient self-ceased).
- Robotic left partial nephrectomy (13 February 2020)—Histopathology: pT1b type 1 papillary RCC.

**Figure 1:**
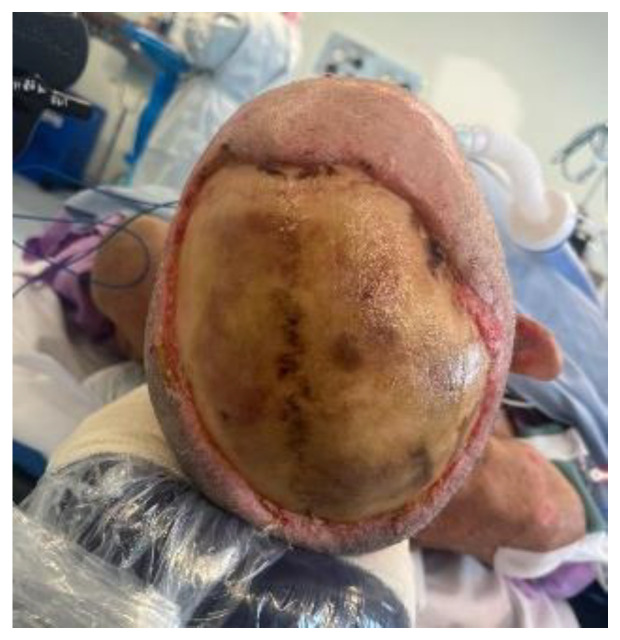
Scalp defect with exposed calvarium, day 13 postinjury.

He presented with nine percent total body surface area (TBSA) burns, with entry at the scalp and exit through the left forearm and right flank. There were mixed depth burns to his face, chest, and abdomen. The scalp wound was full thickness to the calvarium, leaving a defect approximately 13 cm × 8 cm in size (see Figure 1).

There was concern for global weakness at presentation as the neurological examination showed 4/5 power in the bilateral lower limbs and clonus on the right side.

Initial surgical management involved a tangential excision of the burn wound and the application of Biobrane^TM^ to the chest, abdomen, left forearm, and right flank five days post-injury. He returned to the operating theatre one week later for debridement and allograft to the scalp, chest, right flank, and left forearm.

Due to the patient’s poor premorbid nutritional status, the initial scalp reconstruction and skin grafting were delayed to allow for the optimisation of his nutritional reserve.

The patient had serial reassessments and subsequently developed progressive neurological decline over two weeks. The American Spinal Injury Association (ASIA) impairment scale was completed, showing paralysis or a palpable contraction in the lower limb muscle groups and variable motor scores in the upper limb. He demonstrated patchy sensation loss with preservation of anal tone (see Table 1). The index MRI scan of the spine was normal.

**Figure 2:**
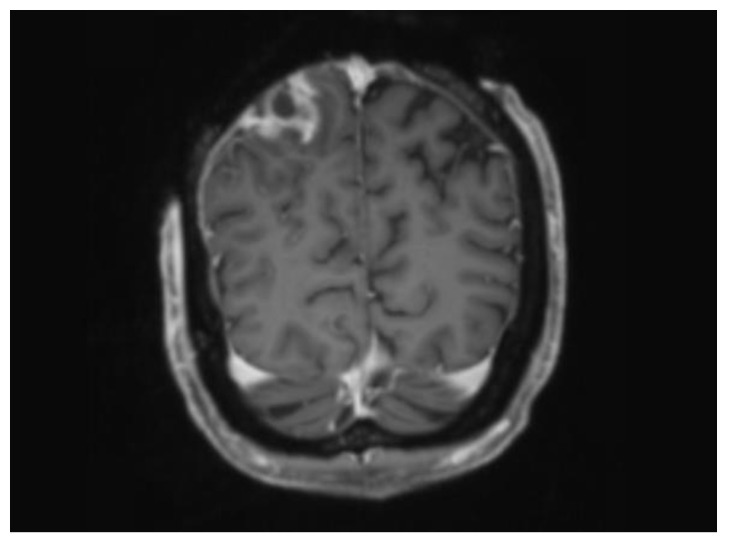
MRI coronal slice demonstrating peripherally enhancing collection interposed between inner table and dura mater.

Twenty-seven days post-injury, the patient had a CT scan for surgical planning to assess the viability of the calvarium. It showed an intact outer table with loss of normal bone marrow signal and suspicion for an underlying extradural abscess, which was an unexpected finding given that the patient was clinically stable. This required collaboration across a number of subspecialty areas including the following: Plastic and Reconstructive Surgery (PRS), Neurosurgery, Neuroradiology, and Burns. An urgent MRI was subsequently arranged, supporting the findings of a peripherally enhancing collection overlying the posterior right parietal lobe (see Figure 2).

**Figure 3:**
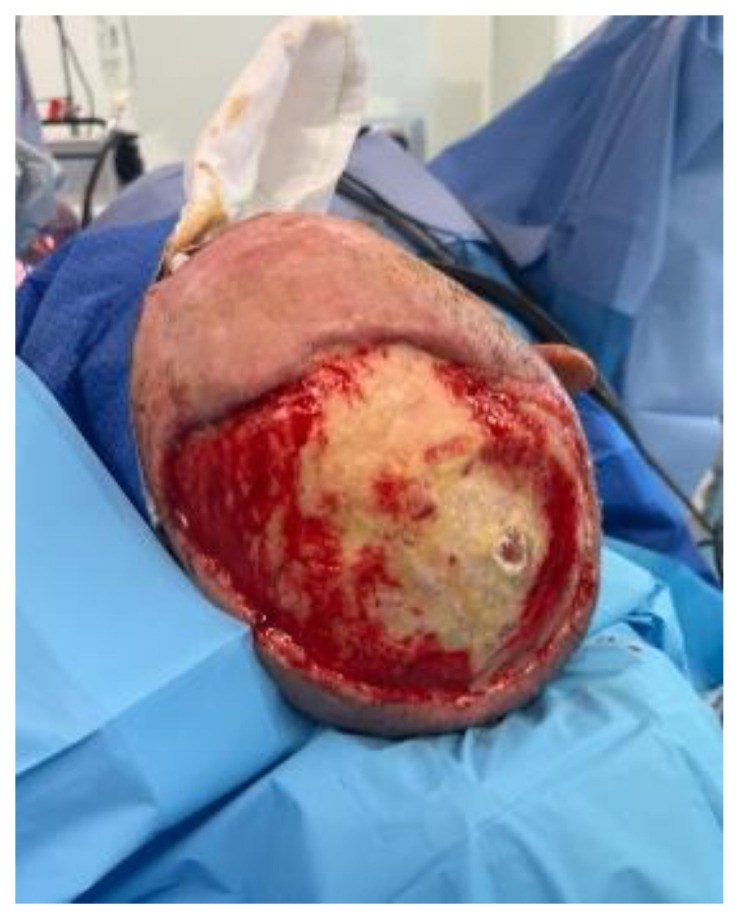
Outer table burred to bleeding diploe. In the right parietal region, there is a 3 cm^2^ defect with dura in base from burr hole.

**Figure 4:**
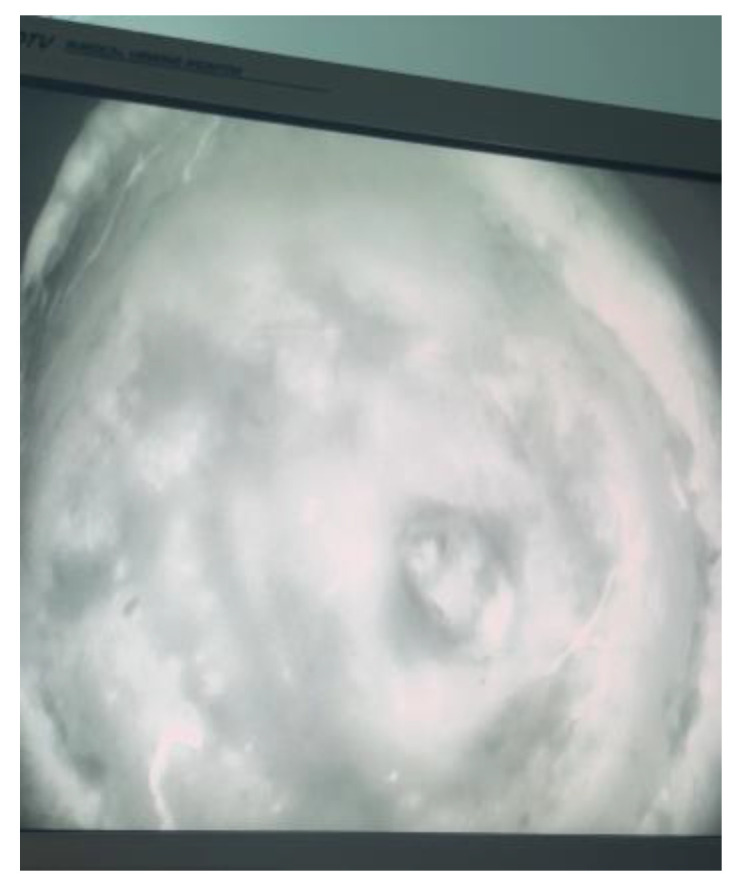
Taken from the SPY-PHI camera showing perfusion to the remaining calvarium.

**Figure 5:**
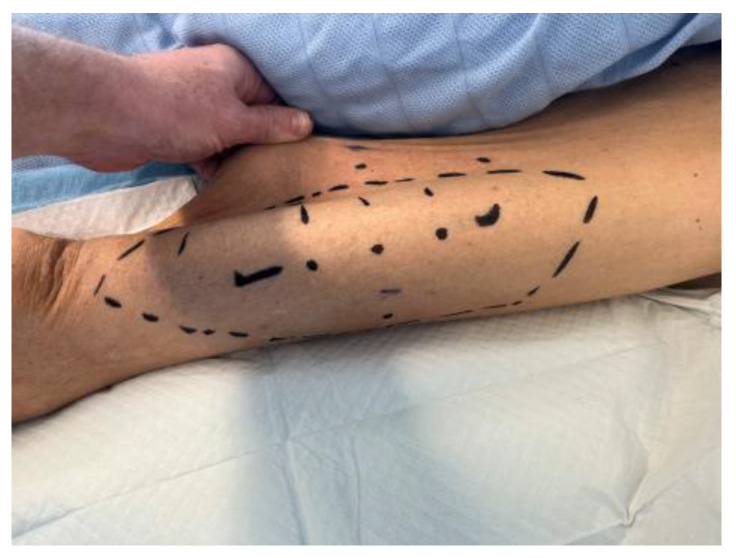
Donor site from the anterolateral thigh.

**Figure 6:**
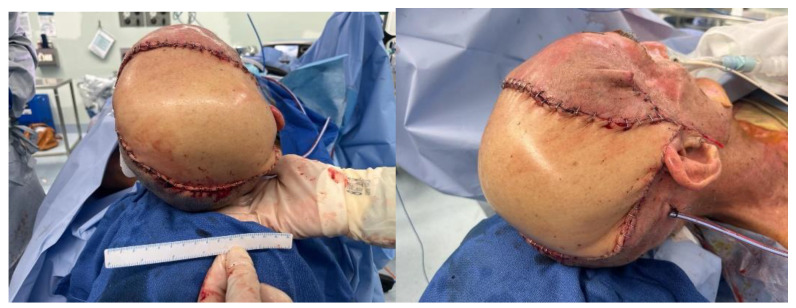
Reconstruction using a free myocutaneous flap from the anterolateral thigh with inclusion of vastus lateralis.

A combined case between PRS and Neurosurgery was planned for the following day. The operation included debridement of the wound edges. Initial drainage of the abscess was carried out with a burr hole under Stealth guidance. Intra-operative findings confirmed pus on the opening of the extradural space. The dura was found to be paper thin with an area of phlegmon covering. Samples were taken for histopathology, microscopy, culture and sensitivity, the dura was subsequently washed with hydrogen peroxide and normal saline. The calvarium was treated by burring devitalised bone to bleeding bone. This necessitated partial thickness resection of the outer table over the majority of the exposed bone; however, there was an area around the burr hole, approximately 6 cm × 7 cm that required the removal of the outer table and diploe (Figure 3). Viability of the remaining bone was assessed using a SPY Portable Handheld Imaging (SPY-PHI) camera (Stryker, Portage, MI, USA) (Figure 4). A free myocutaneous flap was harvested from the anterolateral thigh including the vastus lateralis and skin (Figure 5). Flap transfer was complete following end-to-end anastomosis with the right superficial temporal vessels (see Figure 6).

He was commenced on intravenous Ceftriaxone empirically. The culture grew *Proteus mirabilis* and *Enterococcus casseliflavus* that were sensitive to Ampicillin. Serial CT and MRI scans demonstrated no intracranial re-accumulation. A subcutaneous collection was found on MRI imaging twelve days postoperatively. This was subsequently aspirated under ultrasound with no growth. There were no other complications noted following the procedure.

Repeat MRI imaging of the spine showed symmetric T2 abnormalities within the cervical cord posterolaterally, most likely representing ischaemia in this context.

His final surgery included debridement and split skin grafting of the remaining burn wounds to his chest, forearm, and right flank.

The ASIA assessment was repeated, reporting mild improvement in his lower extremity motor score. The patient continued intensive therapy with Allied Health until his discharge and transfer to rehabilitation. Six months post-injury, he self-discharged from rehabilitation (see Table 1). He is presently living at home with full-time carers, mobilising independently with a wheelchair.

## 3. Discussion

This case demonstrated a unique example of the multidisciplinary and sub-specialty care required to manage the patient’s extradural abscess, burn wounds, and neurological impairment.

The three main points of interest in this case are as follows:

▪ Management of an extra-dural abscess in the context of a large scalp defect, with an intact calvarium exhibiting partial thickness loss of bone viability.
▪ Challenges and surgical timing of tissue coverage given that definitive and urgent treatment of the abscess was required.
▪ The neurological sequelae from his spinal cord injury as a result of the electrical burn.

Following the initial surgical management of his electrical burns, the patient developed delayed neurological deterioration with quadriplegic spinal cord injury and peripheral neuropathy. This was confirmed on MRI imaging demonstrating ischaemic changes throughout the cervical cord. The sensitivity of MRI imaging is variable, especially if performed early but may be useful in describing the expected neurological sequelae [1,10,11]. However, diagnosis should be based on clinical examination [10].

Delayed onset of spinal cord injury is thought to be caused by vascular degeneration or occlusion from vessel wall injury or thrombus, respectively [10,11]. There is an increased risk of ischaemic injury to the anterior grey matter, characteristically resulting in motor nerve dysfunction [5,10]. The pattern of peripheral nerve injury in high-voltage electrical burns appears secondary to axonal injury rather than demyelinating injury [6]. These changes were reflected in the nerve conduction studies completed on the patient two months post-injury, highlighting the mixed aetiology of his neurological symptoms.

In cases where spinal cord injury presents within days to weeks, recovery tends to be prolonged, and prognosis is variable [10]. Predicting the neurological manifestations is difficult; however, the entry/exit sites may offer guidance for future risk assessment in this cohort [5,10].

The incidental finding of an extradural abscess on the pre-operative imaging was of particular interest in this case. This was an unexpected outcome as the patient was asymptomatic, suggesting detection before the clinical symptoms became apparent. His neurological state remained stable during this time.

In the literature, one case study describes a 6-year-old boy who presented with a cerebral abscess two months after an electrical burn to the scalp, which was initially managed with dressings [8]. He suffered cerebral contusions at the time of injury [8]. The patient passed away within 24 h before surgical intervention was attempted [8]. To the authors’ knowledge, there are no other case studies describing the development of an abscess following an electrical burn.

Detection of an intracranial abscess is a life-threatening emergency. The first goal of treatment is to surgically remove the necrotic and purulent material, in combination with antibiotics.

There were a number of considerations in this case including the following:

▪ Presence of an extensive full-thickness scalp defect and need for definitive coverage following the aspiration.
▪ Porosity of the underlying bone affecting access and closure.
▪ Perfusion of the calvarium.
▪ Risk of intracranial re-accumulation post-graft and need for further surgery.

Following drainage, definitive treatment of the skin and bone defects needs to be completed. The reconstructive plan involved an assessment of the calvarium, debridement of the necrotic tissue to bleeding tissue, and a decision on whether a local or free flap was most appropriate. Our preference is to place well-vascularised tissue over exposed bone rather than burring the calvarium to allow for the development of granulation tissue [12,13]. In ideal circumstances, the reconstruction would have been delayed thus optimising the patient’s nutritional status.

The timing of scalp reconstruction was brought forward to allow for a definitive coverage immediately following source control.

In electrical burns, there is a risk of vessel thrombosis surrounding the defect, which affects successful flap transfer both intra- and postoperatively [3,12]. The inner table was burred to bleeding tissue and the superficial temporal vessels were viable. Calvarial perfusion was assessed and confirmed intra-operatively with an Indocyanine Green (ICG) perfusion study. This imaging technique uses a light source to excite the target tissue, combined with a SPY Portable Handheld Imaging (SPY-PHI) camera (Stryker, Portage, MI, USA) [14]. It measures returning fluorescence intensity, providing real time visualisation of tissue perfusion [14]. This modality was used to confirm perfusion of the remaining bone and reduce the risk of osteomyelitis [14]. It was also effective in assessing the perfusion of the very large anterolateral thigh flap.

If the calvarium overlying the abscess required a full-thickness resection, a cranioplasty would have been performed as a delayed procedure to ensure coverage of the brain tissue [3]. This is to reduce the risk of colonisation and infection of the cranioplasty [3].

In view of the abscess and potential for repeated operations, a fasciocutaneous flap from the anterolateral thigh was selected over a myocutaneous latissimus dorsi flap. The addition of the vastus lateralis was deemed preferable to increase vascularity and delivery of antibiotics to the infected bed. Therefore, scalp reconstruction was performed with a chimeric myocutaneous anterolateral thigh flap with inclusion of the vastus lateralis.

The patient’s low body mass index (BMI) and excessive loose skin allowed for a direct closure at the thigh.

## 4. Conclusions

This case report describes the rare occurrence of an extradural abscess following a full-thickness electrical burn to the scalp. A brain abscess is a life-threatening condition requiring emergency management. This case highlights the coordination of the multi-disciplinary care team to deliver treatment within 24 h of diagnosis. Challenges relating to the surgical timing, techniques used to determine perfusion of the calvarium, and the choice of tissue coverage are the salient points in this case. The patient’s concurrent neurological damage has resulted in a poor functional outcome that is likely to persist in the long term.

## Figures and Tables

**Table 1:** Timeline of events.

Time (Days)	Event
0	Initial injury
2	Patient presented to his local hospital and was subsequently transferred to a regional hospital.
3	Admitted to the Burns unit.
4	Initial neurological assessment in the Intensive Care Unit showed 4/5 power in bilateral limbs.
	Normal MRI Brain and Spine.
5	Tangential excision of the burn wound and application of Biobrane^TM^ to the chest, abdomen, left forearm, and right flank.
13	Further debridement and application of allograft to the scalp, chest, right flank, and left forearm.
16	ASIA impairment scale completed. Total motor scores: upper extremity 37/50; lower extremity 7/25 Total sensory scores: light touch 72/112; pin prick 53/92 *
27	CT Head demonstrating loss of bone marrow signal and a peripherally enhancing lesion highly suspicious for an underlying abscess. These findings were consistent with MRI imaging.
28	Drainage of abscess and scalp reconstruction with a free myocutaneous flap from the anterolateral thigh.
40	MRI Brain revealing a superficial fluid filled collection in the subcutaneous tissue over the right parietal lobe. MRI spine showed symmetric T2 abnormalities within the cervical cord posterolaterally.
42	Debridement of remaining burn wound and split skin grafting to his chest, left forearm, and right back.
43	Ultrasound-guided drainage of subcutaneous collection.
47	MRI confirming interval reduction in size of the subcutaneous collection. No evidence of intracranial re-accumulation.
53	Repeat ASIA impairment scale: Total motor scores: upper extremity 36/50; lower extremity 13/25 Total sensory scores: light touch 62/72; pin prick 46/72 **
65	Transferred to a regional hospital for rehabilitation.
169	Patient self-discharged from rehabilitation. He requires full-time carers and mobilises independently in a wheelchair.

* Note: T8-T12 not tested on the left due to burn wound; ** Note: T3-T12 not tested due to dressings.

## Data Availability

No new data was created.

## References

[1] Delgadillo D., Chapman S., Fahrenkopf M.P., Martin M.D. (2017). Acute-Onset Quadriplegia with Recovery after High-Voltage Electrical Injury. Ann. Plast. Surg..

[2] Prodanov S.S., Benkova E.G., Chokoeva A.A. (2018). High-voltage electrical injury: Modified surgical technique for optimal defect closuring of extra-large cranial defect. Dermatol. Ther..

[3] Khalid F.A., Ahmed O.A., Jibran Rabbani M., Saleem M., Amin M., Malik Mujahid A., Younas Mehrose M., Tarar M.N., Shahzad F. (2022). An Algorithm for Reconstruction of Electrical Injuries of the Scalp. Plast. Reconstr. Surg..

[4] Zemaitis M.R., Foris L.A., Lopez R.A., Huecker M.R. (2019). Electrical Injuries [Internet]. Nih.gov. Publishing. https://www.ncbi.nlm.nih.gov/books/NBK448087/.

[5] Ko S.H., Chun W., Kim H.C. (2004). Delayed spinal cord injury following electrical burns: A 7-year experience. Burns.

[6] Kwon K.H., Kim S.H., Minn Y.K. (2014). Electrodiagnostic Study of Peripheral Nerves in High-Voltage Electrical Injury. J. Burn. Care Res..

[7] Krishna D., Khan M.M., Dubepuria R., Cheruvu V.P.R. (2023). Reconstruction of Scalp and Forehead Defects: Options and Strategies. Cureus.

[8] Dewan P., Bhayana S., Nag V. (2021). Cerebral Abscess: A Delayed Complication of Electrical Burns. Indian Pediatr..

[9] Laliberté F., Bloom I., Alexander A.J. (2019). The Critical Role of Nutrition in Facial Plastic Surgery. Facial Plast. Surg. Clin. N. Am..

[10] Abebe M.W., Alem S.E. (2022). Late onset quadriparesis in high voltage electrical burn—A case report. Burns Open.

[11] Koller J., Orságh J. (1989). Delayed neurological sequelae of high-tension electrical burns. Burns.

[12] Jeyakumar P., Hussain A.T., Ahamed A.R. (2018). Reconstruction of Extensive Post–Electric Burn Scalp Defects with Exposed Bones. Ann. Plast. Surg..

[13] Harrison S.H. (1951). Exposure of the skull from burns. Br. J. Plast. Surg..

[14] Gurtner G.C., Jones G.E., Neligan P.C., Newman M.I., Phillips B.T., Sacks J.M., Zenn M.R. (2013). Intraoperative laser angiography using the SPY system: Review of the literature and recommendations for use. Ann. Surg. Innov. Res..

